# Supplementary material for: Iterative improvement in the automatic modular design of robot swarms
Source: PeerJ Comput Sci. 2020 Dec 7;6:e322. doi: 10.7717/peerj-cs.322 (PMC7924708; doi:10.7717/peerj-cs.322)
Supplement: Supplemental Information 3 [file peerj-cs-06-322-s003.zip › argos3/doc/api/standalone/a00479_source.html]

ARGoS: plugins/robots/foot-bot/simulator/footbot\_turret\_entity.h Source File


- Main Page
- Related Pages
- Namespaces
- Classes
- Files

- File List
- File Members

# plugins/robots/foot-bot/simulator/footbot\_turret\_entity.h

Go to the documentation of this file.

```
00001 
00007 #ifndef FOOTBOT_TURRET_ENTITY_H
00008 #define FOOTBOT_TURRET_ENTITY_H
00009 
00010 namespace argos {
00011    class CFootBotTurretEntity;
00012    struct SAnchor;
00013 }
00014 
00015 #include <argos3/core/simulator/entity/entity.h>
00016 #include <argos3/core/utility/math/vector3.h>
00017 
00018 namespace argos {
00019 
00020    class CFootBotTurretEntity : public CEntity {
00021 
00022    public:
00023 
00024       ENABLE_VTABLE();
00025 
00026       enum EMode {
00027          MODE_OFF,
00028          MODE_PASSIVE,
00029          MODE_SPEED_CONTROL,
00030          MODE_POSITION_CONTROL
00031       };
00032 
00033    public:
00034 
00035       CFootBotTurretEntity(CComposableEntity* pc_parent);
00036 
00037       CFootBotTurretEntity(CComposableEntity* pc_parent,
00038                            const std::string& str_id,
00039                            SAnchor& s_anchor);
00040 
00041       virtual void Init(TConfigurationNode& t_tree);
00042       
00043       virtual void Reset();
00044 
00045       virtual void Update();
00046 
00047       inline UInt32 GetMode() const {
00048          return m_unMode;
00049       }
00050 
00051       inline void SetMode(UInt32 un_mode) {
00052          m_unMode = un_mode;
00053       }
00054 
00055       CRadians GetRotation() const;
00056 
00057       Real GetRotationSpeed() const;
00058 
00059       const CRadians& GetDesiredRotation() const;
00060 
00061       Real GetDesiredRotationSpeed() const;
00062 
00063       void SetDesiredRotation(const CRadians& c_rotation);
00064 
00065       void SetDesiredRotationSpeed(Real f_speed);
00066 
00067       virtual std::string GetTypeDescription() const {
00068          return "turret";
00069       }
00070 
00071    private:
00072 
00073       /* Anchor associated to this turret */
00074       SAnchor* m_psAnchor;
00075       /* Operational mode (off, passive, rotation control, speed control */
00076       UInt32 m_unMode;
00077       /* Desired rotation set by the actuator */
00078       CRadians m_cDesRot;
00079       /* Desired rotation speed set by the actuator */
00080       Real m_fDesRotSpeed;
00081       /* Current rotation speed */
00082       Real m_fCurRotSpeed;
00083       /* Rotation at previous time step */
00084       CRadians m_cOldRot;
00085 
00086    };
00087 }
00088 
00089 #endif
```

---

Generated on 10 Jul 2018 for ARGoS by 
 1.6.1
